# Influence of nanoparticles on freezing inside container equipped with fins

**DOI:** 10.1038/s41598-022-18714-7

**Published:** 2022-08-30

**Authors:** Adel Almarashi, Amira M. Hussin, M. Mirparizi, Chunwei Zhang, Hosam A. Saad

**Affiliations:** 1 Department of Mathematics, College of Science, Jazan University, New Campus, Post Box 2097, Jazan, Kingdom of Saudi Arabia; 2 Department of Mathematics, Al-Aflaj College of Science and Humanities Studies, Prince Sattam Bin Abdulaziz University, Al-Aflaj, 710-11912 Kingdom of Saudi Arabia; 3 Department of Mechanical Engineering, University of Yazd, Yazd, Iran; 4 Multidisciplinary Center for Infrastructure Engineering, Shenyang University of Technology, Shenyang, 110870 China; 5 Department of Chemistry, College of Science, Taif University, P.O. Box 11099, Taif, 21944 Kingdom of Saudi Arabia

**Keywords:** Nanoscience and technology, Nanoscale materials

## Abstract

With loading of different shapes of nanoparticles, the solidification speed can be changed which was scrutinized in current work. Although the nanoparticles dispersion can decline the heat capacity, the conduction mode can be improved with such technique and changing the styles of nano-powders can alter the strength of conduction. The velocity terms were neglected in freezing, thus, the main equations include two equations with unsteady form for scalars of solid fraction and temperature. Grid adaption with position of ice front has been considered in simulations utilizing FEM. The upper sinusoidal and inner rectangular walls maintain cold temperature and freezing starts from these regions. Adding nanomaterial can expedite the process around 15.75% (for m = 4.8) and 29.8% (for m = 8.6). Also, utilizing particles with shapes of blade form can augment the freezing rate around 16.69%. The efficacy of *m* on freezing process rises around 4% with elevate of concentration of nanoparticles.

## Introduction

Obtaining the steadiness among the minimum and the maximum heat use, or even generation is an absorbing matter among the heat mechanism specialists^1–5^. Within the previous years, scientists in various fields of analyses were made attempts to develop the heating use. The endeavors contain analyses on the increment of thermal procedure^6–10^, promotion of solar unit applying great efficiency substances^11–15^ and etc. Energy storage mechanisms have a decent technique to that matter. Saving energy can happen as latent heat within the change of substance phases at constant temperature^16–20^. The substances applied for this storage were called PCM. Compared to typical sensible unit, a high magnitude of heat can be saved in very lesser volumes of PCMs^21–25^. Within the discharging and charging of heat, PCM can be installed in tinier temperature gradients^26–30^. Nonetheless, the low heat conductivity of the PCMs is the key disadvantage of PCM substances in the performance of heat mechanisms^31–36^. Therefore, increase in the heat conductivity can be assumed as key parameter for industrial uses^37–43^. Cao et al.^44^ expressed that the paraffin charging rate is soared by the addition of fins. The authors saw that the fins number is a main term for each wall temperature.

Authors of^45^ inquired the impact of kind of substance of extend surface and nanomaterial on the efficiency of solar unit and expressed that the presence of such techniques can considerably increase the melting of PCM. Zeng et al.^46^ inquired the impact of various sets of locations of a hole accumulated with paraffin. They saw that the speed of phase change has increased with changing the location to vertical style. Mehta et al.^47^ presented a comparison between the horizontal and vertical containers and saw that the buoyancy force is operational in the vertical unit within the charging procedure, leading to a charging rate is approximately fixed in comparison to the horizontal units. Usman et al.^48^ inquired various shapes of a thermal sink. They saw that installing fins with various arrangements reduce the most appropriate temperature. This was because of an increment in the efficient heat conductivity. Authors of^49^ inquired the impact of Gr and the aspect ratio on the convection diffusion within freezing. Authors saw that sped of freezing relied on the heat and geometrical terms of mechanism. Chen et al.^50^ inquired the impact of porous media and triangular double fins on charging of a vertical container. They saw that applying triangular fins including porous zone makes melting period decreases about 98%. Researchers of^51^ inquired the charging procedure of the paraffin wax as a PCM inside triangular enclosures and they reported the effective impact of apex angle on unsteady process. Mohamed et al.^52^ inquired RT44HC PCM to find the result of different amounts of wall thermal flux and saw that growing the input power decreases the required time by 42.10 percent.

It was mentioned in various published article that mixing the base PCM with nanoparticles can enahnce the performance. So, to accelerate discharging process within the present container, nanoparticles with two styles have been utilized. The modeling procedure with assumption of neglecting velocity has been derived and such equations were solved via FEM. To portray the efficacy of two active factors, contorus and plots have been reported and required time for solidification have been derived for all cases. Validation for this numerical appraoch was done according to previous article and good accomodation was reported.

## Container with cold curved walls

**Figure 1:**
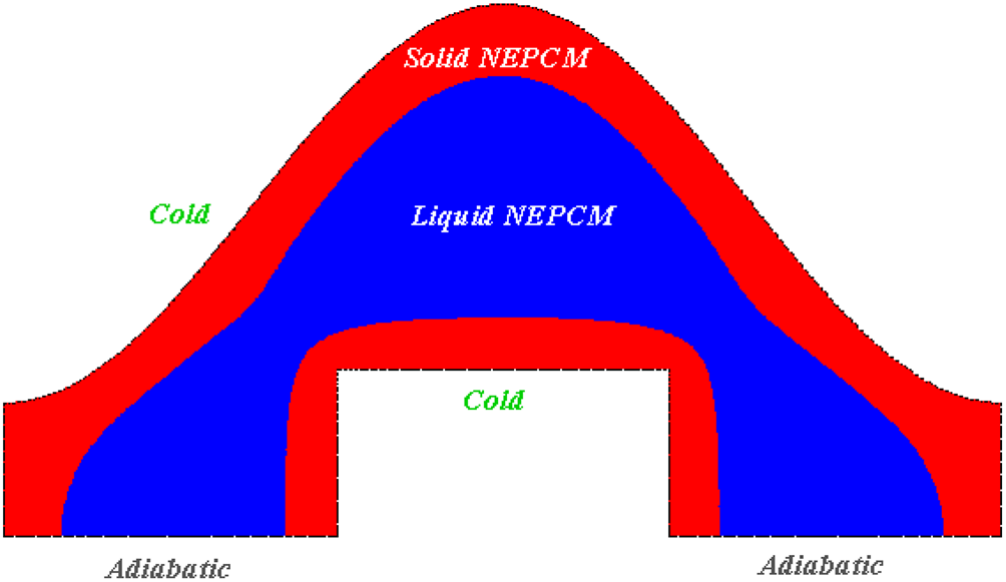
Freezing of water in attendance of nanoparticles through special container.

Two curved walls with shapes of wavy and rectangular have been involved in present study as shown in Fig. 1. The water was mixed with two shapes of nanoparticle (CuO) and to incorporate the modeling, single phase technique was chosen^53^. The curved walls are cold and there exist two adiabatic walls, too. The associated terms of velocity have been discarding in mathematic model because of its low efficacy in freezing. Thus, the related equations for modeling are^53^:

1 $$ \left( {\rho C_{p} } \right)_{nf} \frac{dT}{{dt}} - L_{nf} \frac{dS}{{dt}} = \nabla \left( {k_{nf} \nabla T} \right) $$

2 $$ \begin{aligned} & \left\{ {\begin{array}{*{20}l} {S = 1} \hfill & {AA < \left( { - T_{0} } \right)} \hfill \\ {S = 0} \hfill & {AA > \left( { - T_{0} } \right)} \hfill \\ {S = (T_{m} + 0.5T_{0} - T)/T_{0} } \hfill & {\left( { - T_{0} } \right) < AA < T_{0} } \hfill \\ \end{array} } \right. \\ & AA = \left( {T - T_{m} } \right) \\ \end{aligned} $$

There exist three terms in Eq. (1) which needs to calculate as features of nanomaterial and homogenous mixture approximation has been utilized for this purpose. The below formulas were utilized in current study^53^:

3 $$ \rho_{nf} = \rho_{f} (1 - \phi ) + \rho_{p} \phi $$

4 $$ \left( {L\rho } \right)_{nf} = \left( {L\rho } \right)_{f} (1 - \phi ) $$

5 $$ \frac{{\left( {C_{p} \rho } \right)_{nf} - \left( {C_{p} \rho } \right)_{f} }}{{\left( {\left( {\rho C_{p} } \right)_{p} - \left( {C_{p} \rho } \right)_{f} } \right)}} = \phi $$

6 $$ \frac{{k_{nf} }}{{k_{f} }} = \frac{{ - m\,BB\,\phi + k_{p} + mk_{f} - \phi BB + k_{f} }}{{k_{f} m + k_{f} + BB\,\phi + k_{p} }},\,BB = \left( {k_{f} - k_{p} } \right) $$

In Eq. (6), there is term of shape factor to involve the various shapes of nanoparticles in simulations. Two shapes of cylinder (m = 4.8) and blade (m = 8.6) shapes were incorporated. The transient model need powerful method for modeling especially it should be combined with adaptive grid to increase the accuracy of modeling. Sheikholeslami^53^ suggested to use finite element based approach in modeling of freezing process and He utilized various numerical approaches for designing the thermal storages units. In this work, the same method was utilized with involve of Galerkin method.

## Results and discussion

**Figure 2:**
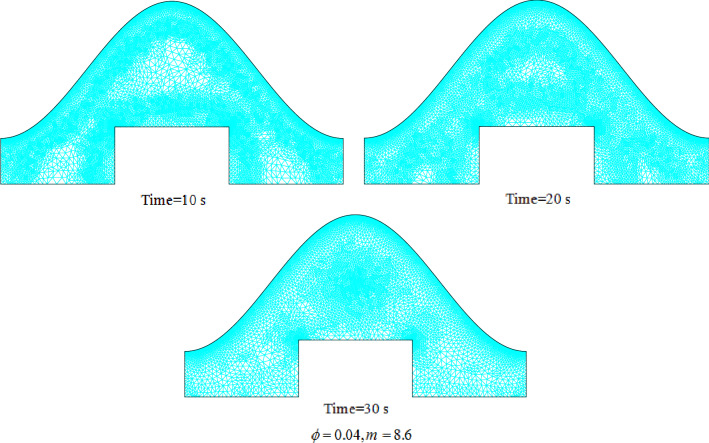
Grid resolution in different stages of process.

**Figure 3:**
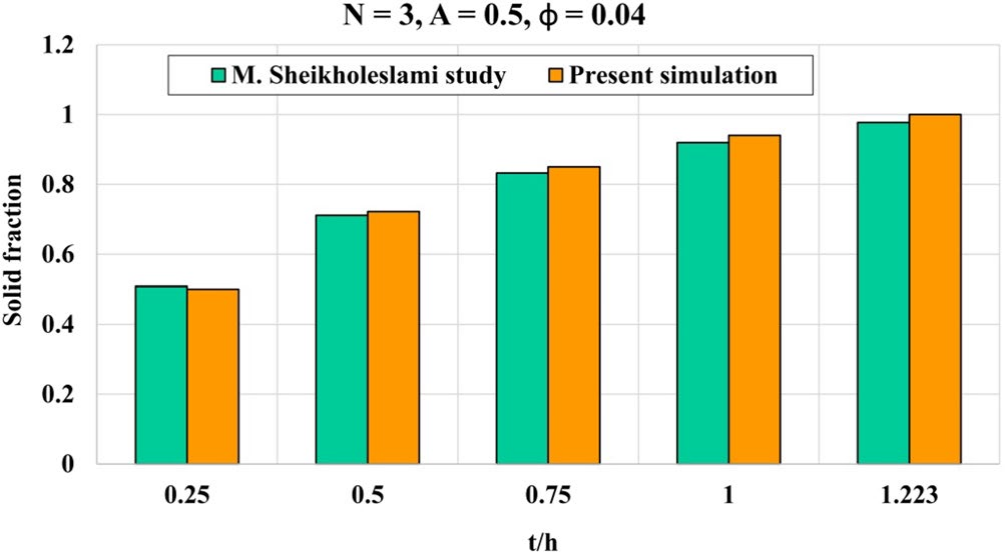
Evaluation of correctness of simulation^53^.

The tank including inner rectangular cylinder connecting to cold flow as well as outer sinusoidal cold wall has been analyzed in current study for heat release phenomena. The tank was filled with liquid water which is mixed with CuO nanoparticles to remove the limitation of its inherent low conductivity. Two different shapes of particles were involved to detect the efficacy of this factor on solidification rate. In scrutinized geometry, there exist two horizontal walls which are adiabatic while other wall has temperature of lower than 273 K. To gain the mathematical model, this fact that there is small value of velocity for freezing process has been considered. Thus, the equations of model include temperature equation and concentration of solid PCM. In temperature equation, the advection terms have been neglected and implicit technique for modeling process has been involved. Also, to include the freezing phenomena, there exit one transient term to represent converting liquid to solid. Efficacy of nanomaterial can be appeared in calculation of features of NEPCM which was done based on homogeneous model. The modeling was done by FEM approach and the style of mesh has been changed with augmenting time. Figure 2 portrays the resolution of grid at three levels of time and indicates the more number of elements have been applied in space near the ice front because the magnitude of temperature gradient is stronger than other places. The previous publication for validation purpose has been analyzed^53^ and related outputs have been demonstrated in Fig. 3 which demonstrates good accommodation. So, the same modeling procedure has been utilized for present problem and single phase approach for modeling of NEPCM treatment was applied.

**Figure 4:**
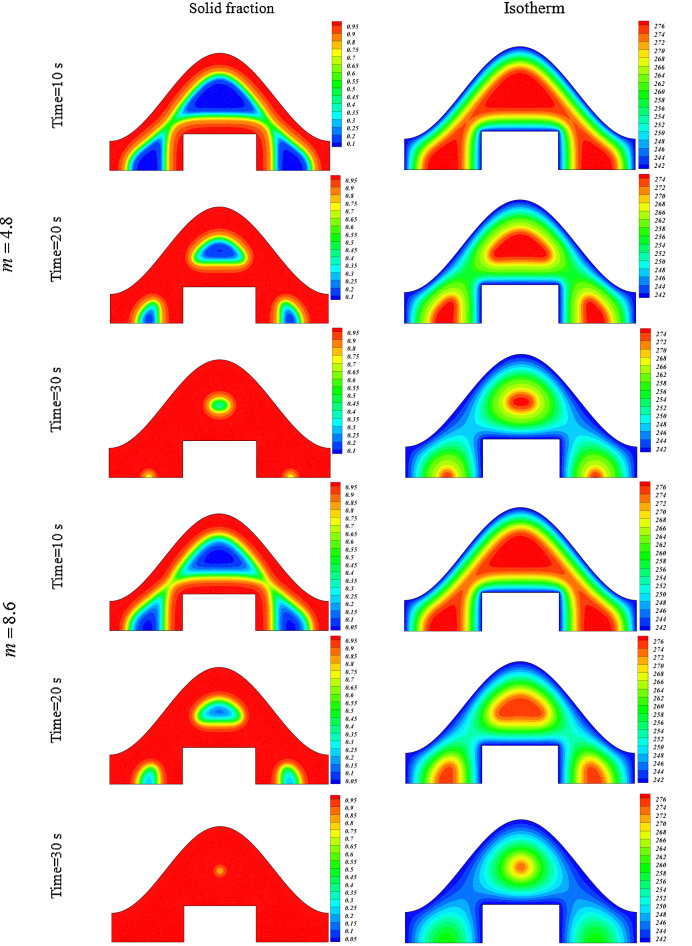
Impact of configuration of nano-powders on transient phenomena.

**Figure 5:**
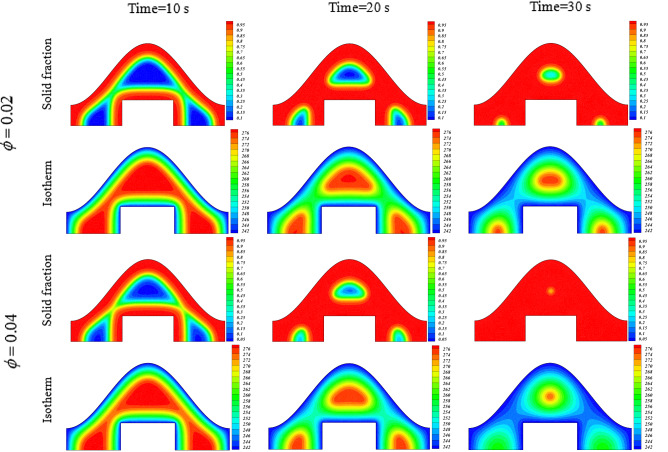
Roles of ϕ on transient phenomena.

In this work, disperse of nanoparticles was applied as main method of enlightening the speed of freezing. Not only was the volume of particles but also configuration of powders assumed as active factor in modeling. Owing to low velocity of liquid phase, the main term in this phenomenon is thermal diffusion term which is main origin of conduction mode. Figures 4 and 5 illustrate the transient behavior of scalars of this work with applying various levels of active factors. The freezing time for pure water case is about 44.71 s and inclusion of nanoparticles with concentration of 0.02 leads to reduce of freezing time up to 40.1 s and 37.66 s for particles with shape factor of 4.8 and 8.6, respectively. Moreover, if the concentration of blade shape particles augments, the period declines from 37.66 to 31.37 s. Increase of shape factor can augment the freezing rate and its amount for ϕ = 0.04 is 4.5% superior than that of ϕ = 0.02. With intensify of ϕ, the speed of process increases and the related value for blade shape is 4% greater than that of m = 4.8.

**Figure 6:**
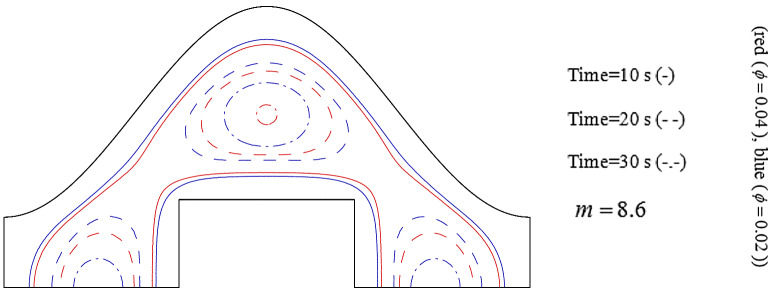
The role of ϕ on movement of ice front.

**Figure 7:**
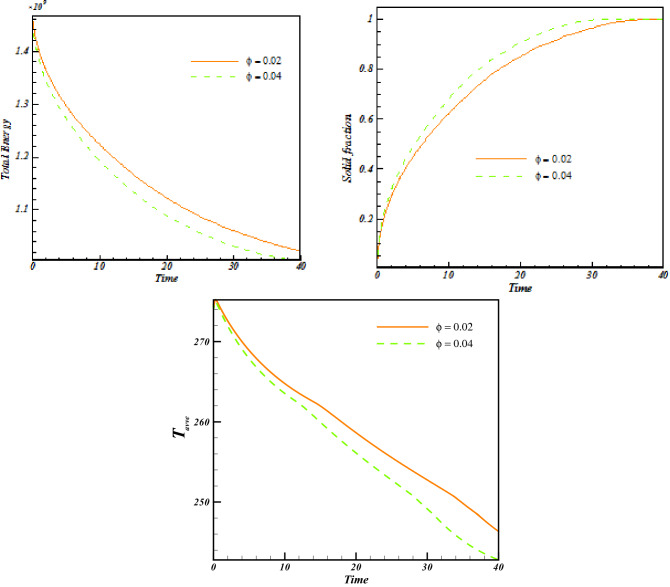
Progress of time change of parameters with later of ϕ.

**Figure 8:**
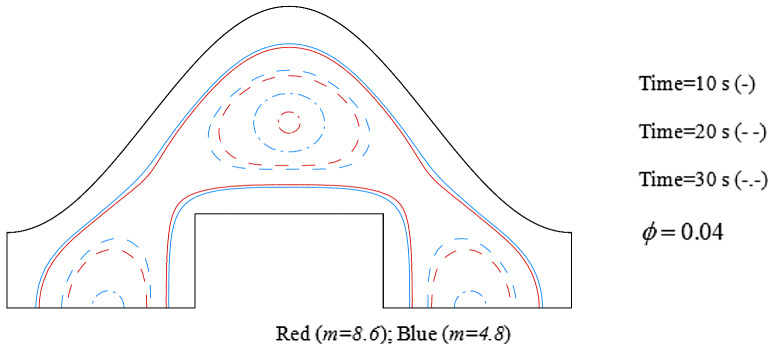
Role of *m* on movement of ice front.

**Figure 9:**
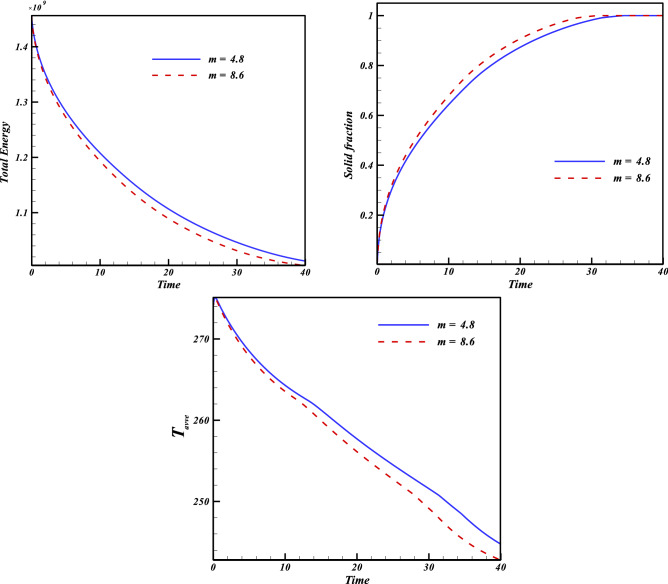
Progress of time change of parameters with later of *m*.

**Figure 10:**
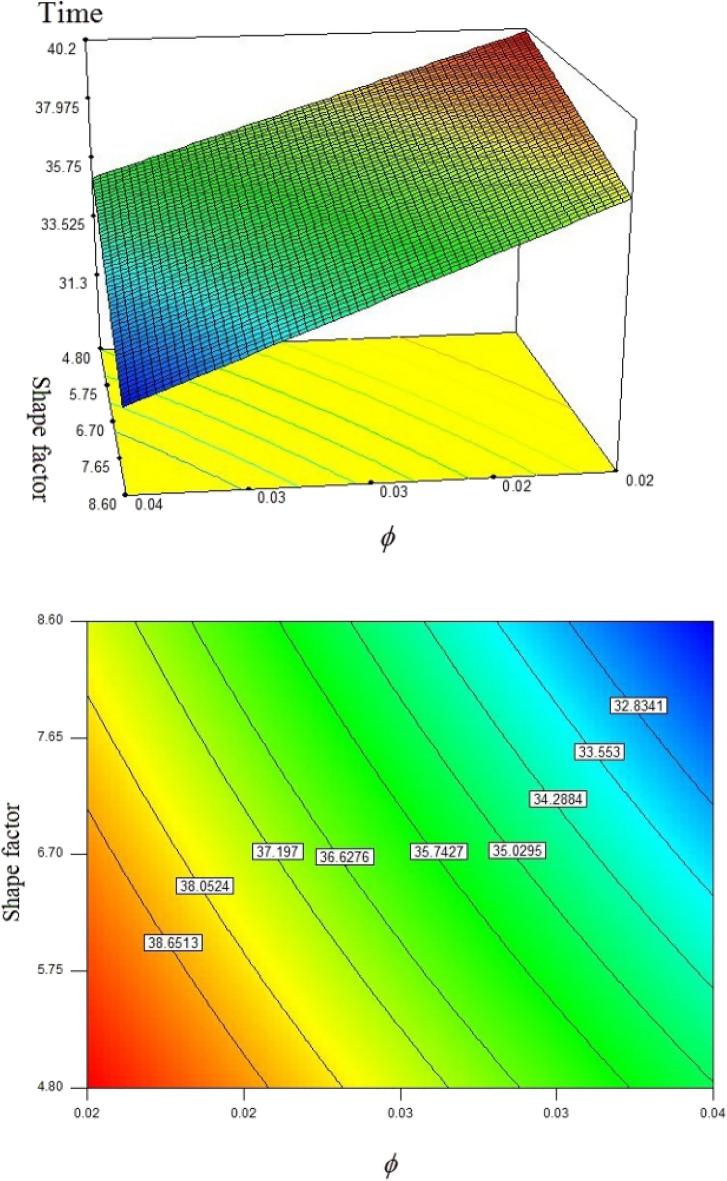
Change of factors and computed period of process.

Increase of ϕ can change the movement of ice front as shown in Fig. 6 and average values of functions can be calculated in each time stages and related data were reported in Fig. 7. Reducing tendency of temperature and energy with progress of time is due to reduction of liquid PCM and more reduction can be observed if concentration of nanomaterial increases. The solid fraction enhances with grow of time and adding nanoparticles makes the magnitude of this scalar to increase. The comparison of blade and cylindrical shapes of nanoparticles in view of ice front and mean value of scalars were portrayed in Figs. 8 and 9. When the all domain convert to solid, the magnitude of SF extents to unity and minimum level of temperature can be reported. Both sensible and latent heat declines with promote of time, thus the energy of unit reduces. The amounts of energy for blade shape particles are lower than the other style of particle because of lower temperature. The significant factor of designing unit for discharging process is period of time and the associated data has been shown in Fig. 10. With augment of concentration of nano-powders with blade and cylindrical shapes, the needed time declines around 12.54% and 16.69%, respectively. Adding nano-powders can augment the freezing rate around 15.75% and 29.81%. Moreover, with change of style of particles from cylinder to blade shapes, the required time decreases around 6.07% and 10.53% when ϕ = 0.02 and 0.04, respectively.

## Conclusion

Inclusion of nano-powders with two different shapes has been considered as passive technique of augmenting speed of phase change phenomena. The container has rectangular and sinusoidal walls which connected to cold flows and their temperature has been considered lower than 273 K. To find the new properties of water after dispersing nanoparticles, homogeneous mixture assumption has been applied. With appearance of nanoparticles, the penetration of cold region augments because of greater conduction mode. FEM technique was applied involving the time-dependent style of grid which helps to model the region of ice front more carefully. To verify the procedure of modeling, previous article has been validated and outputs depicted the good agreement. In calculation of conductivity of produced material, the influence of shape of nano-powders was applied and this factor has significant impact on freezing. The active factors of current study are concentration and shapes of nanoparticles and two levels have been utilized for reach of them. The augment of concentration makes the conduction to increase and penetration of cold flow increases which makes the solidification to accelerate. The temperature of zone reduces with rise of time and involving higher concentration of nanoparticles offers lower temperature. Also, the needed time can decline with soar of ϕ. The cylindrical and blade shapes were incorporated in this work and blade shape has greater conductivity which offers lower time for full solidification. Also, this shape has lower level of energy because of lower temperature of domain. With rise of ϕ with blade and cylindrical styles of particles, the required time decreases around 12.54% and 16.69%. Freezing time declines around 15.75% and 29.81% with adding nanoparticles depends the style of powders. Besides, with alter of style of particles from cylinder to blade shapes, the needed time decreases by 6.07% and 10.53% at ϕ = 0.02 and 0.04. The solidification time for pure water case is about 44.71 s and with adding blade shapes nanoparticles, the needed time reaches to 31.37 s.
